# A novel NFAT1-IL6/JAK/STAT3 signaling pathway related nomogram predicts overall survival in gliomas

**DOI:** 10.1038/s41598-023-38629-1

**Published:** 2023-07-14

**Authors:** Chao Zhang, Yu Wang, Wei Shao, Dongrui Zhou, Dong Yu, Shiqiang Hou, Ning Lin

**Affiliations:** Department of Neurosurgery, The Affliated Chuzhou Hospital of Anhui Medical University, The First People’s Hospital of Chuzhou, Chuzhou, 239000 China

**Keywords:** Biochemistry, Biological techniques, Cancer, Computational biology and bioinformatics, Biomarkers, Diseases, Molecular medicine, Neurology, Oncology

## Abstract

The NFAT1-mediated IL6/JAK-STAT signaling pathway has been observed to contribute to malignant progression in glioma patients. To predict the overall survival (OS) rate of these patients, a prognostic model was developed based on this pathway. Two datasets, mRNAseq_325 and mRNAseq_693, were obtained from the China Glioma Genome Atlas (CGGA), excluding some patients with a lack of survival information, resulting in the inclusion of 684 glioma cases. The two groups were randomly divided into training and validation groups to analyze the differential expression of NFAT1 in pan-cancer and investigate the relationship between differential NFAT1 expression and glioma clinicopathological factors and Transcriptional subtypes. A prediction model based on the IL6/JAK/STAT signaling pathway was constructed using the LASSO-COX dimension reduction analysis to predict the OS of glioma patients. Pearson correlation analysis was utilized to identify gene sets associated with patient risk scores and to perform GO and KEGG analyses. NFAT1 is differentially expressed in a variety of cancers and is enriched in the more malignant potential glioma subtypes. It is an independent prognostic factor in glioma patients, and its expression is significantly positively correlated with the IL6/JAK/STAT signalling pathway in glioma patients. The final prediction model incorporating the seven candidate genes together with other prognostic factors showed strong predictive performance in both the training and validation groups. Risk scores of glioma patients were correlated with processes such as NF-κB and protein synthesis in glioma patients. This individualized prognostic model can be used to predict the OS rate of patients with glioma at 1, 2, 3, 5, and 10 years, providing a reference value for the treatment of glioma patients.

## Introduction

Gliomas are the most natural primary malignant tumors in the central nervous system (CNS) and have high recurrence and mortality rates^1^. Despite various treatment modalities for gliomas, such as surgical resection, chemotherapy, and immunotherapy, it also remains highly unsatisfactory, especially glioblastoma multiforme (GBM). Currently, the primary treatment strategy for gliomas involves surgery, and followed by postoperative adjuvant radiotherapy and chemotherapy^2,3^. Although several prognostic markers and clinical prediction models have been developed, they still fail to meet the requirements of individualized treatment and OS prediction for gliomas. Therefore, it is crucial to establish more practical and accurate prediction models to guide treatment decisions and assess prognosis for gliomas.

Nuclear factor of activated T cell-1 (NFAT1) is a type of protein that is activated by calcium and has important functions in both immune response and neurodevelopment. When calcium levels increased in a cell, the N-terminal homology domain (NHD) domain of NFAT1 binds to calcineurin, which causes NFAT1 to be dephosphorylated and move into the nucleusand regulates the expression of various genes downstream^4^. NFAT proteins have been found to play a role in promoting cancer cell proliferation, inhibiting apoptosis, inducing the invasion and migration of cancer cells, and contributing to drug resistance. These effects can occur through both calcineurin-dependent and calcineurin-independent pathways^5^. NFAT1-regulated IL6 signaling helps increase glioma aggressiveness, emphasizing the function of immunomodulators in deterioration of gliomas^6^. IL6 is a pleiotropic cytokine that can promote T cell activation, B cell differentiation, and modulate acute-phase responses^7,8^. Typically, IL6 binds to its specific receptor IL6R, then, IL6 activates the JAK/STAT pathway by dimerizing the signal-sensor receptor^9^.

Carcinogenic signaling pathways have been identified as important factors contributing to tumor development, with one of the most significant being the Janus tyrosine kinase (JAK) transducer and transcription signal transduction (STAT) activator pathway^9^. This pathway is related to mediating cell replication, anti-apoptosis, angiogenesis, and immunosuppression-related functions in the tumor microenvironment^10,11^, making it a major source of tumor progression and drug resistance^12^. The JAK/STAT signaling pathway could facilitate the conversion of signals from outside the cell into internal physiological processes^13^. By controlling the transcription of certain target genes, this pathway regulates cell growth and differentiation^14^. Dysregulation of this pathway is associated with some tumors, as JAK/STAT pathway genes promote the growth, proliferation, survival, inflammation, invasion, angiogenesis, and progression of various tumors, including breast, prostate, glioblastoma^15,16^.

Previous studies have demonstrated that the JAK/STAT signaling pathway involved in the progression, migration, and invasion of glioblastomas. IL-8 is produced in the tumor microenvironment, which activates STAT1 to promote tumor migration, invasion, and mesenchymal transformation^17^. Additionally, STAT5 is associated with tumorigenesis and can promote tumor proliferation and invasion^18,19^. In glioblastoma patients, the levels of STAT3 and IL-6 correlate with clinical stage of the tumor, forming constitutive activation^20^. NFAT1 regulates IL6 signaling pathway, and the RORA-mediated NDRG2-IL6/JAK/STST3 signaling pathway can lead to invasive behavior of gliomas and promote glioma cell proliferation^6,22^. Therefore, blocking these signaling pathways is seen as a potential treatment for gliomas. In this study, we identified NFAT1 as an independent prognostic factor for glioma patients by conducting differential expression analysis of NFAT1 in pan-cancer and investigating its role in malignant progression in glioma patients. Furthermore, we constructed risk profiles for glioma patients based on the NFAT1-IL6/JAK/STST3 signaling pathway. Using mRNAseq_325 as the training group and mRNAseq_693 as the validation group, we explored the biological properties associated with glioma prognosis and established a glioma prognostic model. This model can more accurately assess the prognosis of glioma patients and provide an effective tool for guiding their treatment.

## Result

### NFAT1 is enriched in pan-cancer and glioma malignant subtypes

**Figure 1:**
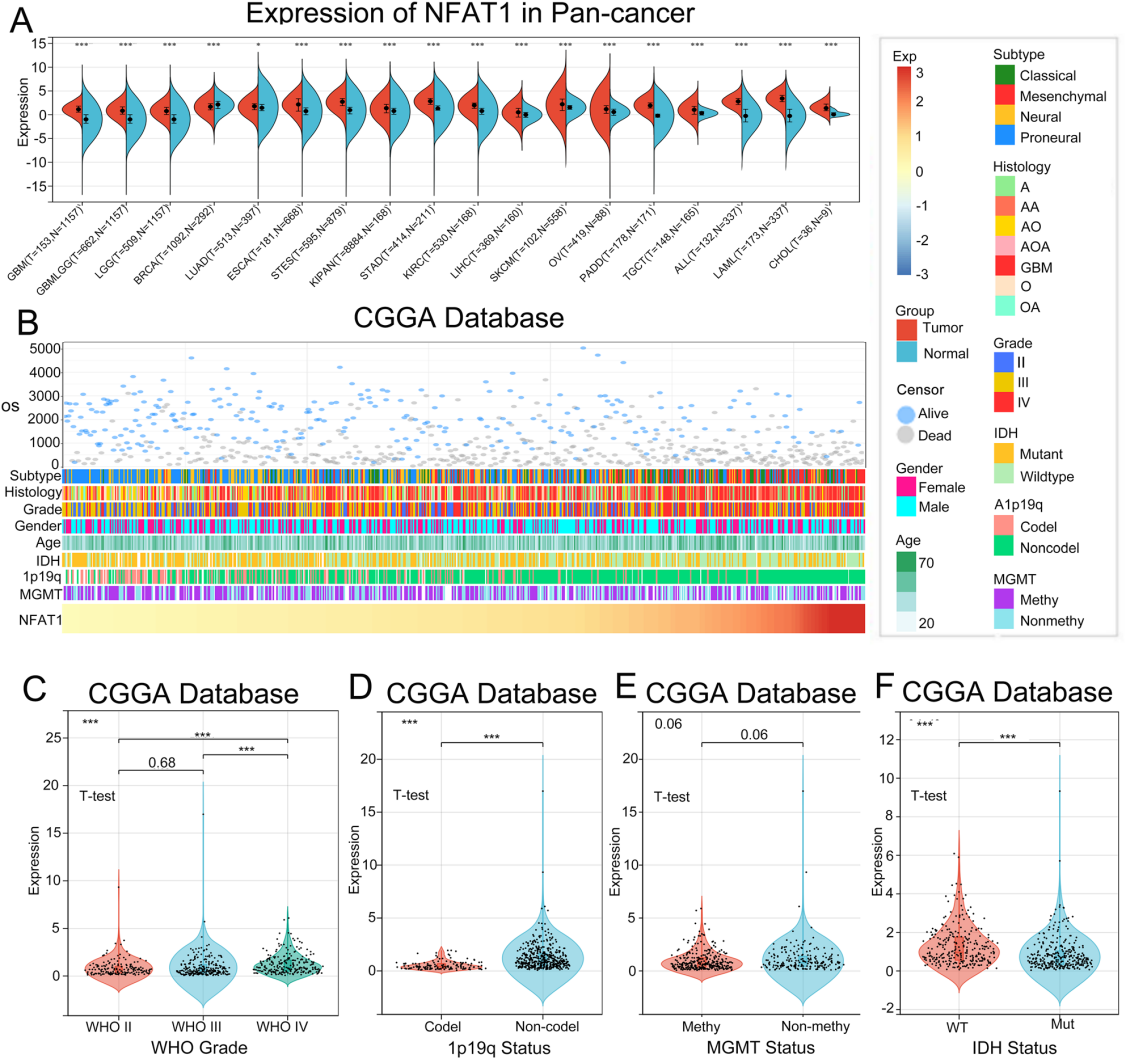
Differential expression of NFAT1 in pan-cancer and glioma versus the normal tissue. (**A**) Differences in NFAT1 expression between pan-cancer and normal tissues. (**B**) The expression of NFAT1 showed an asymmetric trend between the clinicopathological factors in glioma patients. (**C–F**) The violin plot shows significant enrichment of NFAT1 in the malignant subtype of glioma. The significance of the difference was tested using an unpaired T test.

**Figure 2:**
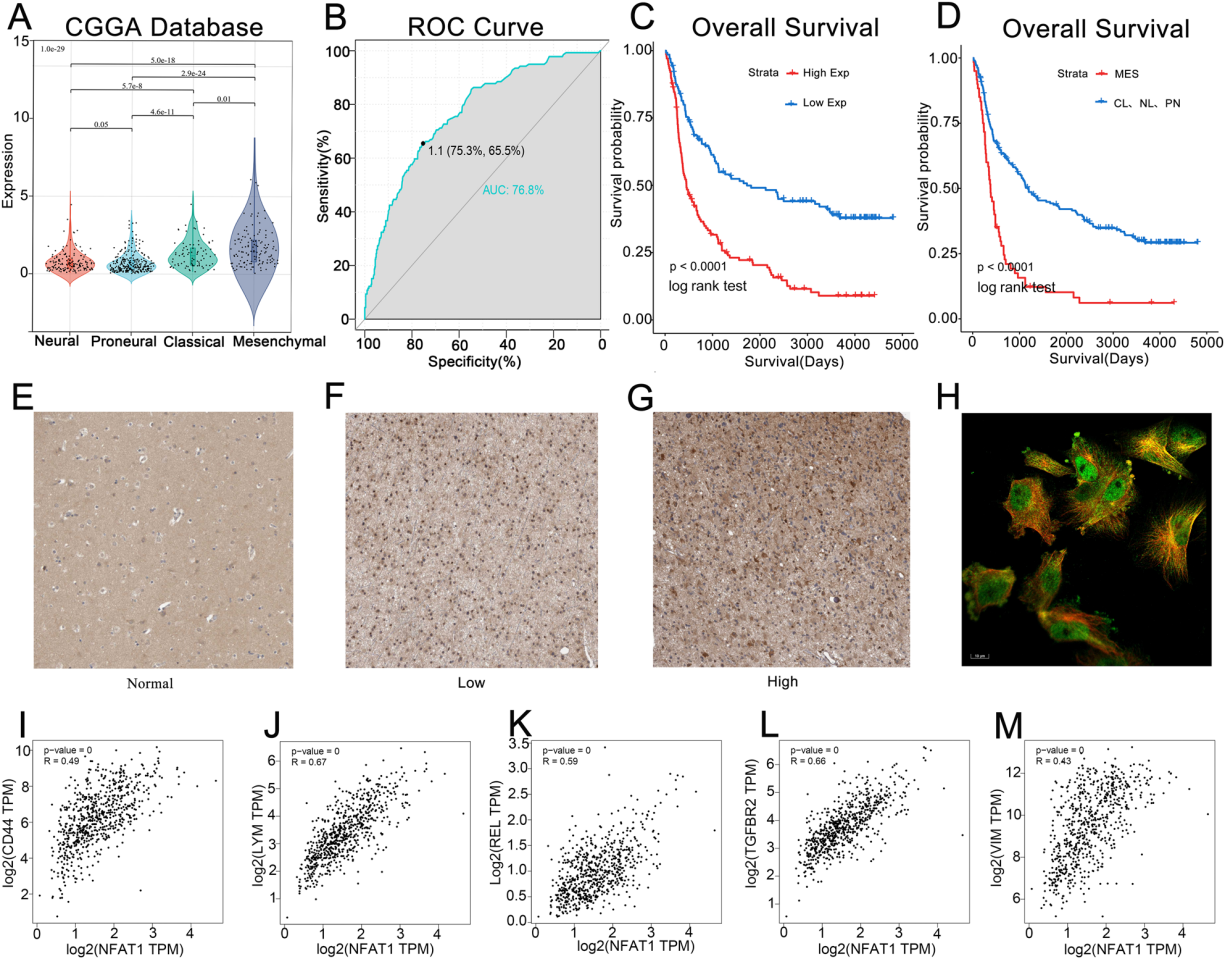
Association between NFAT1 expression and glioma mesenchymal subtypes. (**A**) NFAT1 is enriched in mesenchymal subtypes of gliomas with higher malignant potential. The significance of the difference was tested by one‐way ANOVA. (**B**) ROC curves showed high expression specificity of NFAT1 in glioma mesenchymal subtypes. (**C**) Patients of gliomas with high NFAT1 expression show poor prognosis. (**D**) The prognosis of glioma patients with mesenchymal subtype is worse than other subtypes. The significance of the prognostic value was tested by a log‐rank test. *MES* mesenchymal, *CL* classic, *NL* neural type, *PN* proneural type. (**E**) NFAT1 expression level in normal brain tissue. (**F**) Brain tissue expression of NFAT1 in patients with low-grade gliomas. (**G**) Brain tissue expression of NFAT1 in patients with high-grade gliomas. (**H**) The green fluorescence in the immunofluorescence stained picture of the U-251 MG represents the localization of NFAT1 in the nucleoplasm as well as in the cytoplasm of the cells. (I-M) NFAT1 expression was significantly correlated with CD44 (R = 0.49, P < 0.05), VIM (R = 0.43, P < 0.05), REL (R = 0.59, P < 0.05), LYN (R = 0.67, P < 0.05) and TGFBR2 (R = 0.66, P < 0.05).

Firstly, we compared the expression levels of NFAT1 in tumour tissues with their corresponding normal tissues or peritumoural normal tissues. The expression level of NFAT1 in normal tissues or peritumoural normal tissues corresponding to the tumour tissue was considered as the control group. Further analysis of the relationship between differential NFAT1 expression in gliomas and clinicopathological factors and OS. Based on the relevant information provided in the TCGA database, we found that NFAT1 was highly expressed in a variety of cancers, such as ovarian, lung and esophageal cancers, but the expression of NFAT1 was lower in breast cancer than in normal tissues (Fig. 1A). Further analysis using glioma-related information in the CGGA database showed that patients with different NFAT1 expression levels exhibited varying clinical and molecular pathological features. The study found that WHO grade, 1p/19q codeletion status, MGMT promoter methylation status, and IDH mutation status showed an asymmetric distribution in the CGGA database (Fig. 1B). NFAT1 expression was positively correlated with glioma WHO grade (Fig. 1C), where samples without 1p/19q codeletion demonstrated higher levels of NFAT1 expression (Fig. 2D). Additionally, NFAT1 expression was higher in IDH wild-type patients (Fig. 1F). NFAT1 was highly expressed in samples without MGMT promoter methylation, although not statistically significant (Fig. 1E). Taken together, these results suggest that NFAT1 is enriched in gliomas with a higher degree of malignancy.

### NFAT1 differential expression is associated with glioma mesenchymal transformation

Gliomas in the CGGA database have been classified into four transcriptional subtypes, with the mesenchymal subtype exhibiting higher NFAT1 expression levels than the classical, neural, and proneural subtypes (Fig. 2A). The specificity of NFAT1 expression in the glioma mesenchymal subtype was evaluated using the ROC curve, which showed an area under the curve (AUC) of 76.8% in the CGGA database (p < 0.0001) (Fig. 2B). Furthermore, high NFAT1 expression levels in gliomas were associated with poor prognosis (Fig. 2C). Compared to other glioma subtypes, the mesenchymal subtype is more aggressive and radiologically resistant, leading to a worse prognosis (Fig. 2D). To confirm NFAT1 gene expression in clinical samples, the Human Protein Atlas (HPA) demonstrated the expression of NFAT1 in normal brain tissue and glioma tissue, with higher expression levels of NFAT1 observed in higher-grade gliomas (Fig. 2E–G). Moreover, NFAT1 was localized in the Nucleoplasm and cytoplasm of the human astrocytoma cell line (U-251 MG) (Fig. 2H). Mesenchymal transformation is related to the acquisition of stem genes^21^, and our investigation revealed that NFAT1 expression was significantly correlated with stem genes (CD44 and VIM) and mesenchymal transition-related genes (REL, LYN, and TGFBR2) (Fig. 2I–M).

### Personalized prediction models based on IL6/JAK/STAT signaling pathways show unique accuracy

**Figure 3:**
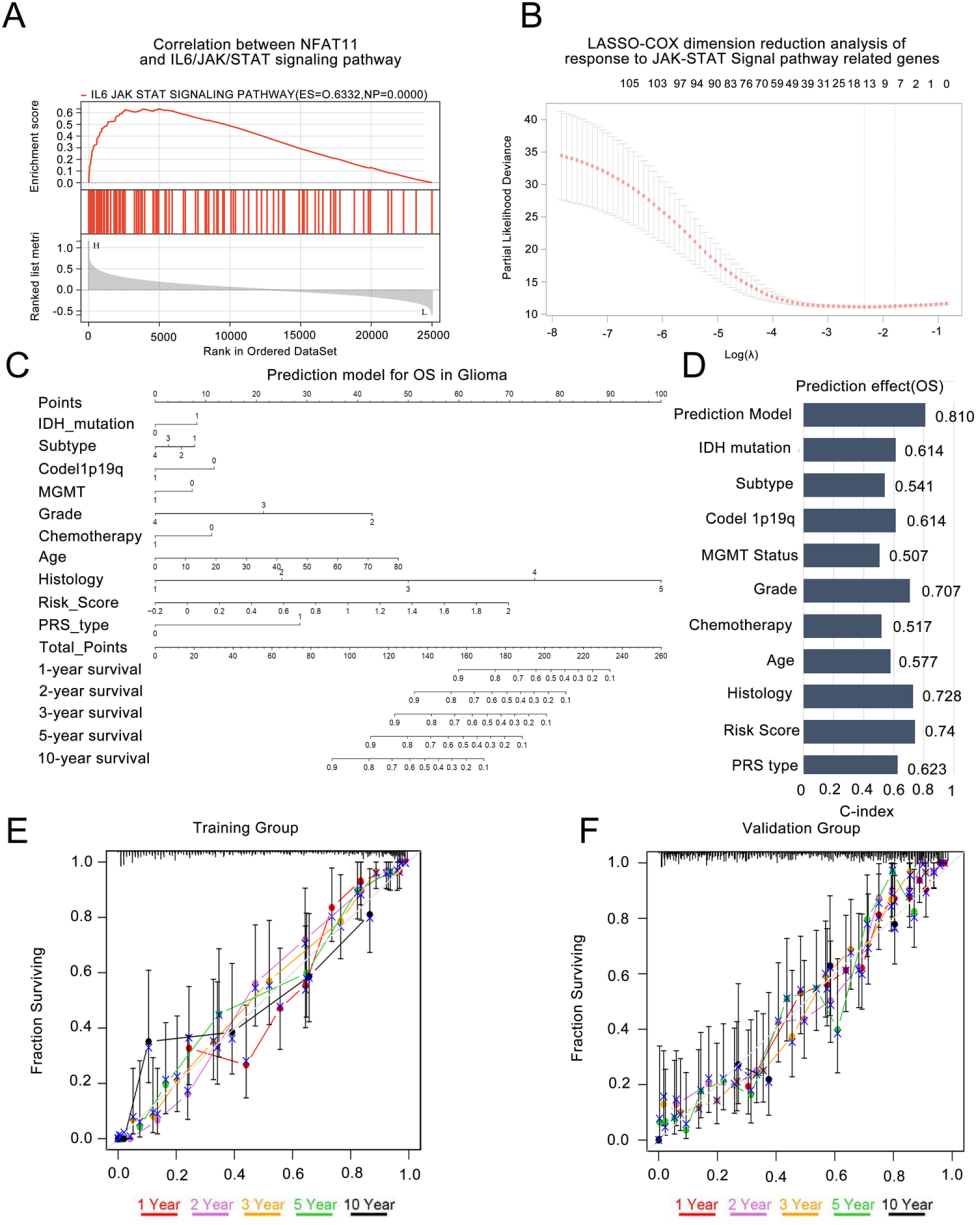
An individualized predictive model of OS in glioma patients. (**A**) NFAT1 expression and IL6/JAK/STAT signaling pathway were significantly correlated in CGGA database. (**B**) Seven most representative genes of IL6/JAK/STAT signaling pathway were screened by LASSO-COX analysis. (**C**) Nomogram accurately predicts the OS at 1, 2, 3, 5 and 10 years after surgery in glioma patients. (**D**) As part of the individualized prediction model, the C index was used to evaluate the prediction effect of risk score and various clinical prognostic factors on OS. (**E,F**) Calibration plots show predicted and actual OS comparisons about 1, 2, 3, 5 and 10 years about the training and validation groups.

To facilitate the clinical application of prediction models based on the CGGA database, we investigated the relationship between NFAT1 and the IL6/JAK/STAT signaling pathway. We constructed an individualized prediction model based on this pathway (Fig. 3A). We used LASSO-COX analysis to construct risk profiles of genes associated with NFAT1-related IL6/JAK/STAT signaling pathways in gliomas, screened for genes associated with glioma prognosis, and retained seven genes based on optimal λ values (PIK3CB, SOS2, PIK3R1, IFNGR2, STAT3, PIK3R3 and IL10RB) (Fig. 3B). We calculated the total risk score using the following formula: Risk score (Table 1) = (− 0.0104 × PIK3CB exp) + (− 0.0047 × SOS2 exp) + (− 0.0022 × PIK3R1 exp) + (0.0006 × IFNGR2 exp) + (0.0062 × STAT3 exp) + (0.0152 × PIK3R3 exp) + (0.0378 × IL10RB exp). We then used multifactorial COX dimension reduction analysis to screen for independent prognostic factors and construct an individualized prediction model to predict OS in glioma patients. The factors included IDH mutation status, different transcriptional subtypes, 1p/19q codeletion status, WHO grade, MGMT methylation status, chemotherapy or not, age, pathological classification, and risk score. In this study, a nomogram of ten prognostic factors in glioma patients was constructed to predict patient OS at 1, 2, 3, 5 and 10 years. In the nomogram, patient OS was estimated by adding up the total number of points obtained for each prognostic factor (Fig. 3C). Our personalized prediction model had a C index of 0.810 (95% CI 0.785, 0.835) in the training group and 0.786 (95% CI 0.761, 0.811) in the validation group, which was an improvement over other current prediction models (Fig. 3D). The calibration curve and actual observation results showed satisfactory results in the training group and the verification group, indicating that the personalized prediction model we constructed was accurate and has clinical application value (Fig. 3E,F).

**Table 1:** Lambda values of included genes.

Included genes	Lambda (λ)
PIK3CB	− 0.0104
SOS2	− 0.0047
PIK3R1	− 0.0022
IFNGR2	0.0006
STAT3	0.0062
PIK3R3	0.0152
IL10RB	0.0378

### Establish and evaluate risk profiles associated with IL6/JAK/STAT signaling pathways

**Figure 4:**
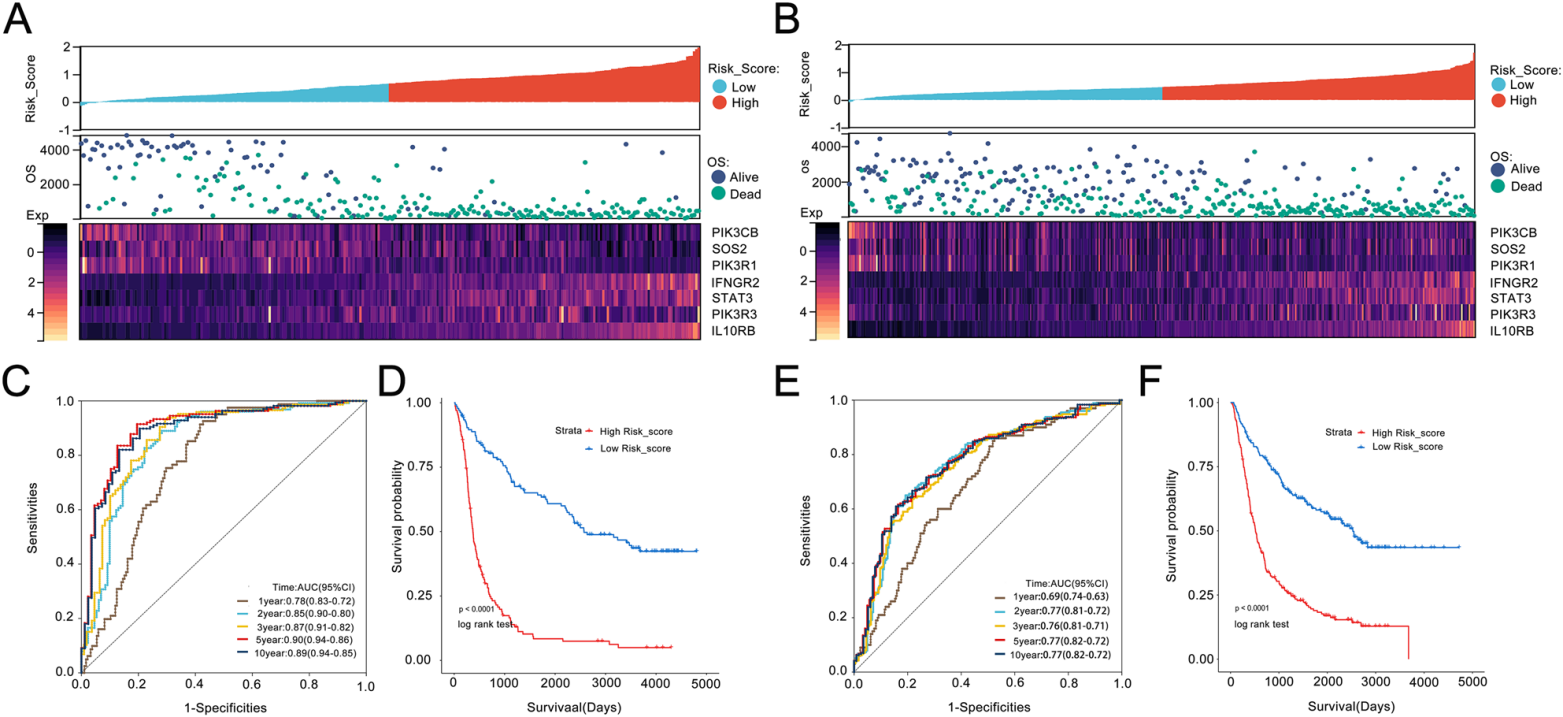
Relationship between risk scores and molecular characteristics and overall OS in glioma patients. (**A,B**) The heatmap showed the difference in candidate gene expression for each glioma patient when the risk scores for the high-risk and low-risk groups are arranged in ascending order, and the data are all TPM (Transcripts Per Million) normalised. (**C,E**) The ROC curves indicates that risk scores showed better predictive accuracy in glioma patients. (**D,F**) According to Kaplan–Meier curves, OS was shorter in high-risk groups. The significance of the prognostic value was tested by a log‐rank test.

The glioma patients were categorized into high-risk and low-risk groups based on the median risk score of each cohort. As the risk score increased, patients in different risk groups displayed varying molecular signatures associated with the IL6/JAK/STAT signaling pathway (Fig. 4A). These findings underscore the precision of predicting outcomes in glioma patients based on risk profiles linked to the IL6/JAK/STAT signaling pathway. Notably, the risk score exhibited prognostic significance in gliomas even with different risk cut-offs. Moreover, risk scores can function as independent prognostic factors, with Kaplan–Meier curves demonstrating better outcomes in the low-risk group among glioma patients (Fig. 4D). ROC curves were utilized to evaluate the predictive value of prognostic models constructed using IL6/JAK/STAT pathway genes. The AUC for predicting OS of 1, 2, 3, 5, and 10 years between glioma patients in the CGGA database were 0.78, 0.85, 0.87, 0.90, and 0.89, respectively (Fig. 4C). The AUC for predicting OS at 1, 2, 3, 5, and 10 years for glioma patients in the training group were 0.78, 0.85, 0.87, 0.90, and 0.89, respectively (Fig. 4E). The differential expression of genes between the high and low-risk groups in the validation group was also evident (Fig. 4B), and the AUC also showed good predictive value, with the Kaplan–Meier curve indicating a shorter OS in the high-risk group compared to the low-risk group (Fig. 4F).

### Risk scores are associated with positive regulation of NF-κB signaling pathway and protein synthesis

**Figure 5:**
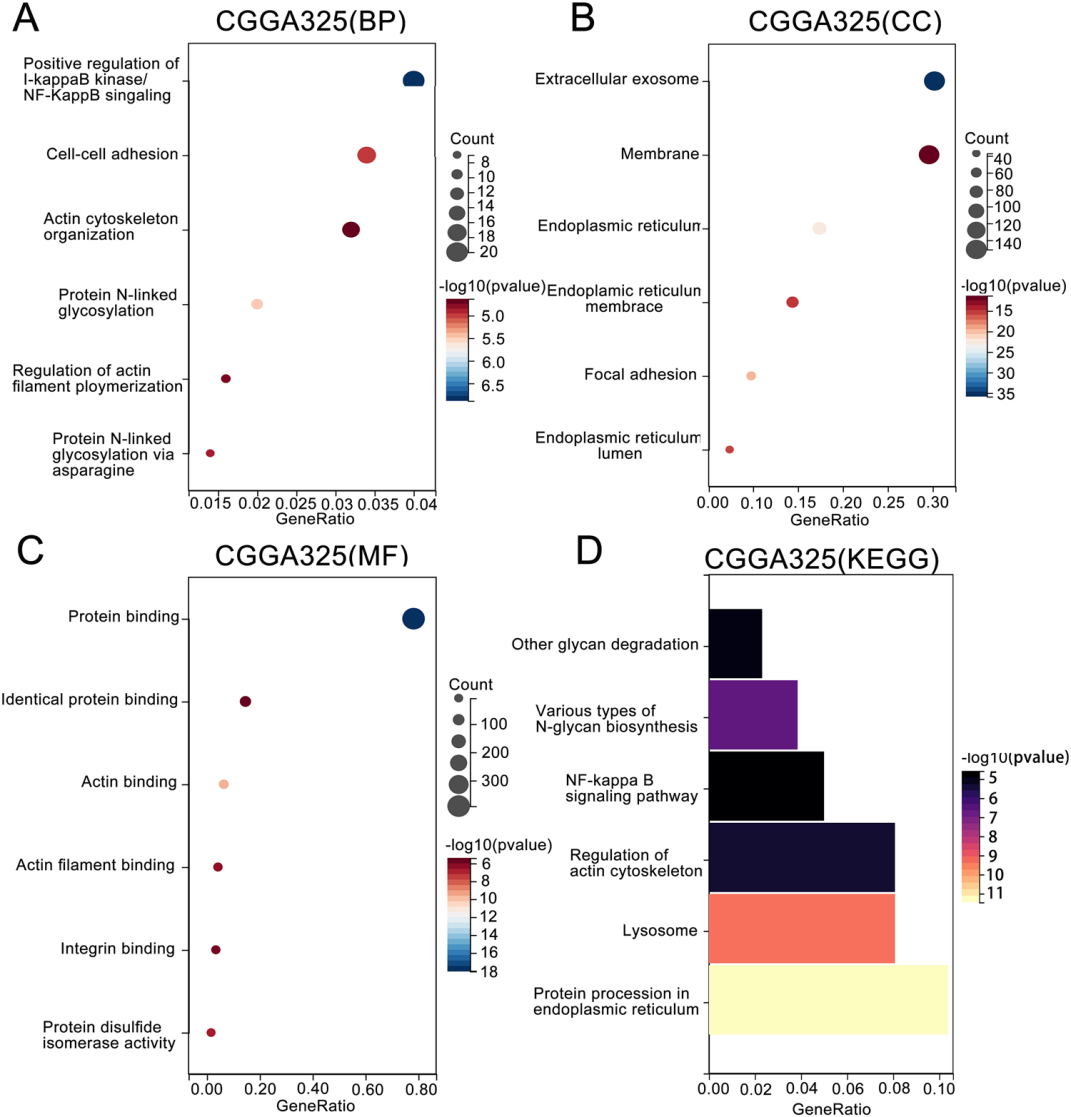
The biological functions and pathways associated with risk scores. (**A–D**) GO and KEGG functional enrichment analysis were performed on the risk score-related gene sets in the CGGA database. The results showed that the biological processes and pathways related with risk scores were related to the positive regulation of NF-κB signaling, cell adhesion, and protein synthesis.

To analyze the biological functions associated with risk scores in glioma patients, we screened the 500 genes most strongly associated with risk scores using Pearson correlation analysis (|R| > 0.6, p < 0.05), and GO and KEGG analyses were performed based on the above gene sets. GO analysis revealed that the biological processes most strongly associated with risk scores included positive regulation of the NF-κB signaling pathway, cell–cell adhesion, and actin cytoskeleton organization (Fig. 5A). Additionally, the cellular components most strongly associated with risk scores were extracellular exosomes and membranes (Fig. 5B). The molecular functions were related to protein binding, including identical protein binding, actin binding, and actin filament binding (Fig. 5C). KEGG analysis showed that the signaling pathways most strongly associated with risk scores in glioma patients were Protein processing in the endoplasmic reticulum and Various types of N-glycan biosynthesis (Fig. 5D). NF-κB is related to viral infection, immune response, cell proliferation, and tumor formation^23^. Activation of the NF-κB signaling pathway in tumor cells is related to pathogenesis and chemotherapy resistance. Inhibition of NF-κB is widely considered a powerful therapeutic approach for inhibiting the proliferation and survival of glioma tumor cells^24^, which may partly explain why the prognosis of glioma patients in high-risk groups is worse.

## Discussion

Glioma is a highly aggressive tumor with poor clinical outcomes, particularly for GBM, which has a severe malignant degree. GBM consists of heterogeneous tumor cells, including poorly differentiated glioma stem cells, which are considered important factors in promoting therapeutic resistance and tumor recurrence^25^. As a result, the prognosis of gliomas has not significantly improved despite the use of surgery combined with radiotherapy, immunotherapy, and gene therapy. Therefore, the development of accurate prognostic models can undoubtedly provide clinical decision-making solutions for precision medicine follow-up of glioma patients. In this study, we identified seven candidate genes associated with glioma prognosis based on the NFAT1-IL6/JAK/STAT signaling pathway to calculate risk scores. Univariate and multivariate analyses were performed to screen for factors of prognostic value, such as IDH mutation status, 1p19q status, MGMT methylation status, chemotherapy usage, WHO classification, transcriptional subtype, and pathological subtype. All factors with prognostic value were combined with risk scores to construct a prediction model, which showed good predictive performance in both the training and validation groups.

Immunoinflammatory factors play a role in the occurrence and malignant progression of gliomas. While the inflammatory environment may precede the development of gliomas, these tumors can then exploit the expression of inflammatory mediators to create a new immune microenvironment that promotes proliferation and migration^26^. There is evidence that gliomas downregulate immune-related protective genes and upregulate cancer-causing inflammatory factors, thereby enhancing their survival^27^. NFAT1 is an essential regulatory transcription factor for the immune system that controls the expression of various cytokines and their receptors^28^. NFAT1 is an integrator of multiple signaling pathways and a core gene that regulates the inflammatory response. Many aspects of cancer may be affected by NFAT1, such as Proliferation, invasion, metastasis, anti-apoptosis^29^. The present study found similar results, showing that NFAT1 is highly expressed in various tumors, including gliomas, and is more pronounced in higher grade gliomas. Furthermore, the study revealed that high NFAT1 expression may play a role in promoting glioma mesenchymal transition.

Several solid tumors and hematologic malignancies have been associated with the activation of JAK/STAT signaling^30^. Additionally, STAT3 has been implicated in tumor progression in glioblastoma^31^. In this study, we found a positive correlation between NFAT1 expression in glioma and JAK/STAT signaling pathway activation. The activation of STAT2 signaling also contributes to the self-renewal of glioblastoma stem-like cells and promotes tumorigenesis. Silencing STAT3 can inhibit glioma cell invasion and growth^32^. STAT3 is a key driving factor for the invasive growth of glioma, making it a potential target for glioma therapy^33^. A inhibitor of the JAK2/STAT3 pathway called WP1193 could induce glioma cell cycle arrest and apoptosis^34^. NFAT1 regulates signaling in IL6, contributing to the aggressive phenotype of gliomas^6^. Moreover, NDRG2-IL6/JAK2/STAT3 signaling inhibits growth in gliomas and induces apoptosis^22^. Based on the NFAT1-IL-6/JAK/STAT signalling pathway, a prognostic model for glioma was developed, which showed good predictive performance in both the training and validation groups. Meanwhile, the investigator’s laboratory is working on regulating the JAK/STAT signaling pathway by NFAT1 in glioma, with the aim of providing a reference value for the therapeutic research on glioma.

In this study, we leveraged the advantages of the CGGA database to develop and validate a predictive model for OS based on glioma patients with long-term follow-up. Importantly, we discovered a significant relationship between NFAT1 and the IL6/JAK/STAT pathway in glioma, suggesting that NFAT1 could serve as an independent prognostic factor in glioma. We established and verified the accuracy of the prognostic model based on this pathway. The user-friendly nomogram developed in this study can be effectively utilized as a tool to personalize prognosis prediction for glioma patients and guide their long-term treatment.

However, despite the strengths of our study, there are still some limitations that need to be acknowledged. The limited sample size in the CGGA database may have hindered the establishment of a robust model. With the continuous development of modern medicine, model predictors will need to be continuously refined to achieve high-precision predictions. Additionally, we recognize the need to develop a simpler and more personalized prognostic model that can be easily understood by untrained physicians, patients’ families, and individuals.

## Conclusion

In this study, we have discovered that NFAT1 is expressed differently in various types of cancers and plays a crucial role in malignant progression through the IL6-JAK-STAT3 signaling pathway in glioma patients. Moreover, we have developed a glioma OS prediction model based on the NFAT1-IL6-JAK-STAT3 signaling pathway. This model can offer practical clinical decisions for personalized treatment of glioma patients to a certain extent.

## Methods

### Data acquisition and download

In this study, the RNA-seq data and clinical data from the China Glioma Genome Atlas (CGGA: http://www.cgga.org.cn/) database were used. This included mRNAseq_325 and mRNAseq_693. Patients with missing survival data and clinicopathological factors were excluded, and 273 patients in mRNAseq_325 were randomly selected as the training group, while 411 patients in mRNAseq_693 were used as the validation group. A total of 684 patients with glioma were included in this study (Table 2). The gene list for the JAK/STAT signaling pathway was obtained from AmiGO2 (http://amigo.geneontology.org/).

**Table 2:** Clinical information of patients.

Characteristic	Subcategory	Training group Total (%) (N = 273)		Validation group Total (%) (N = 411)	
		No	%	No	%
Age	Mean ± SD	43.6 ± 12.2	100.0	43.0 ± 12.4	100.00
Gender	Male	168	61.5	230	55.9
	Female	105	38.4	181	44.0
Grade	WHO II	81	29.6	94	22.8
	WHO III	65	23.8	161	39.1
	WHO IV	127	46.5	156	37.9
IDH_status	Wildtype	130	47.7	184	44.7
	Mutant	143	52.3	227	55.3
1p19q_status	codel	54	19.7	87	21.1
	Non-codel	219	80.3	324	78.8
MGMT_status	methylated	142	52.0	243	59.1
	un-methylated	131	47.9	168	40.9
Subtype	Classical	65	23.8	56	13.6
	Neural	63	23.0	101	24.5
	Proneural	85	31.1	161	39.1
	Mesenchymal	60	21.9	93	22.6

Number of patients enrolled in our study was listed.

### LASSO-COX analysis

We conducted LASSO-COX dimension reduction analysis using the glmnet and survival packages in R software (version 4.1.0). In our study, we selected lambda lse as the optimal lambda value. Based on the OS of glioma patients in the training group, we identified seven candidate genes and their corresponding lambda values. The risk score for each patient was calculated by adding the mRNA-seq values for each candidate gene and multiplying them by their corresponding lambda.

### Nomogram construction

The LASSO-COX dimension reduction analysis was utilized to identify prognosis-related genes in glioma patients, based on the NFAT1-regulated IL6/JAK/STAT signaling pathway. Furthermore, univariate and multivariate analyses were integrated to identify independent prognostic factors in glioma patients and develop a nomogram. A total score was calculated for each glioma patient based on the variables included in the nomogram, with the majority of patients in this study having a total risk score of 260 or less. Based on the median expression level of the prognosis-related risk score, patients were stratified into high-risk and low-risk groups. Kaplan–Meier (K–M) survival analysis was then performed using a survival kit to assess the association between the risk score level and OS (p < 0.05).

### Predictive model validation

We used the ROC curve as the primary measure to assess the predictive ability of our model. The AUC ranges from 0.5 to 1, with higher values indicating better predictive ability. In addition, we utilized the calibration curve and C-index to verify the accuracy and reliability of the nomogram.

### Functional enrichment analysis

We utilized Pearson correlation to analyze the correlation between each glioma patient’s risk score, based on the IL6/JAK/STAT signaling pathway association, and the expression of all genes provided in the database. The top 500 high-risk genes, which were most correlated with the patient’s risk score, were selected based on correlation coefficients. All selected genes had correlations greater than 0.6 and p-values less than 0.001. The list of genes was then uploaded to the DAVID database (https://david.ncifcrf.gov/), where official gene names and Homo sapiens were selected to obtain the results of GO and KEGG analyses, revealing relevant biological processes (BPs), molecular functions (MFs), cellular components (CCs), as well as pathways. In this study, only six significant results at p < 0.05 were observed.

### Immunohistochemistry (IHC)

To further analyze NFAT1 protein differential expression in normal and different pathological grades tissue, we acquired immunohistochemical (IHC) and immunofluorescence images at HPA (http://www.proteinatlas.org/). The antibody HPA024369 was utilized for the IHC staining.

### Statistical analysis

We performed all analyses and visualizations of graphs using statistical software R (version 4.1.0) and SPSS (version 23.0). ROC curves, heatmaps, nomograms, and calibration plots were generated using the R language. Differences were assessed using t-tests, log-rank tests, and one-way ANOVA. Univariate and multivariate Cox proportional hazard regressions were employed to adjust for potential confounding variables. Statistical significance was considered at p < 0.05 for all statistical methods.

## Supplementary Information

Supplementary Information 1.

Supplementary Information 2.

## Data Availability

Publicly available datasets were analyzed in this study. This data can be found here: TCGA: https://portal.gdc.cancer.gov/; CGGA: http://www.cgga.org.cn/; AmiGO2: http://amigo.geneontology.org/; HPA: http://www.proteinatlas.org/.

## Abbreviations

OS: Overall survival
CGGA: The Chinese Glioma Genome Atlas
TCGA: The cancer genome atlas
GO: Gene Ontology
KEGG: Kyoto encyclopedia of genes and genomes
ROC: Receiver operating characteristic
CNS: Central nervous system
GBM: Glioblastoma multiforme
NFAT1: Nuclear factor of activated T cell-1
NDH: N-Terminal NFAT homology domain
AUC: Area under the curve
HPA: Human protein atlas protein atlas
U-251 MG: The human astrocytoma cell line
